# Thirty-First Announcement of the Medical Department of the University of Louisville

**Published:** 1867-07

**Authors:** 


### *Thirty-First Announcement of the Medical Department of the University of Louisville.*

We are pleased to see that this Institution has emerged from its late troubles, and is again before the public, with an able Faculty of representative medical gentlemen, whose names cannot fail to place the Institution side by side, not only with the best on this continent, but of Europe also.

We recognise among the Faculty, the names of some of the ablest medical philosophers of this country,—men, whose education, love of science, and earnest zeal, eminently fit them for the positions they hold; and who will give their great talents, with true fidelity, to teaching, not only the way to health, and how to preserve it, but the necessity of unfaltering devotion, unyielding labor, and self-sacrifice, to carry forward to a glorious consummation the tide of progress which alone can redeem the profession from the wholesale quackery that has threatened it so long.

It is with pleasure that we note the union of the Kentucky School of Medicine with the medical department of the University, thus ending a rivalry disadvantageous, if not discreditable, to both; and we are also pleased to note that some of the faculty of the Kentucky constitute a part of the faculty of the University School. This is as it should be. "Let there be no strife between us;" but with an eye single to the great law of progress, and the greatest good to humanity, go forward with an unbroken front in the career of development and discovery, until ignorant pretension and quackery shall not have a foothold left to stand upon.

---

DR. L. P. YANDELL, JR., a distinguished physician of Louisville, Kentucky, is now in Europe, whither he has gone to prosecute the further study of his profession.

